# Supplementary material for: Pyruvate Dehydrogenase Kinase 1 inhibition mediated oxidative phosphorylation enhancement in cartilage promotes osteoarthritis progression
Source: BMC Musculoskelet Disord. 2023 Jul 20;24:597. doi: 10.1186/s12891-023-06585-6 (PMC10357736; doi:10.1186/s12891-023-06585-6)
Supplement: Supplementary file 1 — Additional file 1. [file 12891_2023_6585_MOESM1_ESM.zip › MF.Gsea.1653627316242/GOMF_PROTEIN_PHOSPHATASE_1_BINDING.html]

Details for gene set GOMF\_PROTEIN\_PHOSPHATASE\_1\_BINDING[GSEA]

|  || Dataset | 55457\_collapsed\_to\_symbols.cls.cls #OA\_versus\_normal.cls.cls #OA\_versus\_normal\_repos |
| Phenotype | cls.cls#OA\_versus\_normal\_repos |
| Upregulated in class | OA |
| GeneSet | GOMF\_PROTEIN\_PHOSPHATASE\_1\_BINDING |
| Enrichment Score (ES) | 0.38016334 |
| Normalized Enrichment Score (NES) | 1.1980228 |
| Nominal p-value | 0.2304038 |
| FDR q-value | 0.54513574 |
| FWER p-Value | 1.0 |
Table: GSEA Results Summary

  

Fig 1: Enrichment plot: GOMF\_PROTEIN\_PHOSPHATASE\_1\_BINDING      
 Profile of the Running ES Score & Positions of GeneSet Members on the Rank Ordered List

  

| SYMBOL | TITLE | RANK IN GENE LIST | RANK METRIC SCORE | RUNNING ES | CORE ENRICHMENT || 1 | PPP1R3C | protein phosphatase 1 regulatory subunit 3C [Source:HGNC Symbol;Acc:HGNC:9293] | 41 | 1.008 | 0.1113 | Yes |
| 2 | KCNQ1 | potassium voltage-gated channel subfamily Q member 1 [Source:HGNC Symbol;Acc:HGNC:6294] | 391 | 0.709 | 0.1647 | Yes |
| 3 | PPP1CA | protein phosphatase 1 catalytic subunit alpha [Source:HGNC Symbol;Acc:HGNC:9281] | 603 | 0.622 | 0.2190 | Yes |
| 4 | PPP1R3D | protein phosphatase 1 regulatory subunit 3D [Source:HGNC Symbol;Acc:HGNC:9294] | 708 | 0.591 | 0.2780 | Yes |
| 5 | PPP1CC | protein phosphatase 1 catalytic subunit gamma [Source:HGNC Symbol;Acc:HGNC:9283] | 744 | 0.583 | 0.3414 | Yes |
| 6 | AKAP11 | A-kinase anchoring protein 11 [Source:HGNC Symbol;Acc:HGNC:369] | 1002 | 0.517 | 0.3802 | Yes |
| 7 | PPP1R3A | protein phosphatase 1 regulatory subunit 3A [Source:HGNC Symbol;Acc:HGNC:9291] | 2732 | 0.277 | 0.2774 | No |
| 8 | PPP1R11 | protein phosphatase 1 regulatory inhibitor subunit 11 [Source:HGNC Symbol;Acc:HGNC:9285] | 3239 | 0.227 | 0.2638 | No |
| 9 | LILRB1 | leukocyte immunoglobulin like receptor B1 [Source:HGNC Symbol;Acc:HGNC:6605] | 5387 | 0.041 | 0.1019 | No |
| 10 | PPP1R16B | protein phosphatase 1 regulatory subunit 16B [Source:HGNC Symbol;Acc:HGNC:15850] | 6283 | -0.026 | 0.0353 | No |
| 11 | STAU1 | staufen double-stranded RNA binding protein 1 [Source:HGNC Symbol;Acc:HGNC:11370] | 7418 | -0.112 | -0.0399 | No |
| 12 | PHACTR4 | phosphatase and actin regulator 4 [Source:HGNC Symbol;Acc:HGNC:25793] | 7674 | -0.133 | -0.0447 | No |
| 13 | LILRB2 | leukocyte immunoglobulin like receptor B2 [Source:HGNC Symbol;Acc:HGNC:6606] | 9317 | -0.257 | -0.1430 | No |
| 14 | C20orf27 | chromosome 20 open reading frame 27 [Source:HGNC Symbol;Acc:HGNC:15873] | 9415 | -0.266 | -0.1204 | No |
| 15 | SHOC2 | SHOC2 leucine rich repeat scaffold protein [Source:HGNC Symbol;Acc:HGNC:15454] | 11440 | -0.494 | -0.2214 | No |
| 16 | FER | FER tyrosine kinase [Source:HGNC Symbol;Acc:HGNC:3655] | 12101 | -0.620 | -0.2022 | No |
| 17 | PPP1R15A | protein phosphatase 1 regulatory subunit 15A [Source:HGNC Symbol;Acc:HGNC:14375] | 12107 | -0.622 | -0.1319 | No |
| 18 | PPP1R10 | protein phosphatase 1 regulatory subunit 10 [Source:HGNC Symbol;Acc:HGNC:9284] | 12568 | -0.780 | -0.0791 | No |
| 19 | PHACTR1 | phosphatase and actin regulator 1 [Source:HGNC Symbol;Acc:HGNC:20990] | 12760 | -0.923 | 0.0109 | No |
Table: GSEA details [plain text format]

  

Fig 2: GOMF\_PROTEIN\_PHOSPHATASE\_1\_BINDING      
 Blue-Pink O' Gram in the Space of the Analyzed GeneSet

  

Fig 3: GOMF\_PROTEIN\_PHOSPHATASE\_1\_BINDING: Random ES distribution      
 Gene set null distribution of ES for **GOMF\_PROTEIN\_PHOSPHATASE\_1\_BINDING**
